# Perceptions of paramedic educators on assessments used in the first year of a paramedic programme: a qualitative exploration

**DOI:** 10.1186/s12909-023-04930-w

**Published:** 2023-12-12

**Authors:** Shane Knox, Charles Brand, Catherine Sweeney

**Affiliations:** 1 National Ambulance Service College – Ireland, The Rivers Building, Belgard Square West, Dublin 24, D24 XNP2 Tallaght Cross, Ireland; 2 Medical Education Unit, University College Cork, Brookfield Health Sciences Complex, Cork, Ireland

**Keywords:** Paramedic, Training, Education, Regulatory oversight, Simulation

## Abstract

**Background:**

In Ireland, there are currently three educational institutions (recognised institutions- RIs) providing paramedic programmes, accredited by the regulator, the Pre-Hospital Emergency Care Council (PHECC). Each RI assesses their students in-house, and in order to acquire a licence to practice, students must also pass summative assessments provided by PHECC. These assessments comprise multiple choice questions, short answer questions and skills assessments. The objective of this study was to explore the perceptions and experiences of paramedic educators of assessments used within their institution and by the regulator to provide insights that could inform the future design of paramedic assessments.

**Methods:**

A qualitative study with an interpretivist approach and purposive sampling strategy was performed. Semi-structured interviews were conducted with educators from one RI, across their three sites. Data were analysed using an inductive approach to thematic analysis.

**Results:**

Four major themes were identified in the data: improving assessments by enhancing authenticity, modifying the current process of assessment, aligning the PHECC and RI examinations and opportunities to use assessment as learning.

**Conclusions:**

This study identifies perceived deficits and opportunities in the assessments currently used for paramedic students and ways in which these assessments could be improved. While participants were relatively content with their own RI assessments, they identified ways to improve both the RI and PHECC assessments. Modifying some of the current methods could be a useful first step. In particular, assessments used by PHECC could be improved by reflecting ‘real-world’ practice. The inclusion of additional assessment methods by PHECC, a continuous assessment process or devolvement of the entire assessment suite, to the RI/University has the potential to enhance assessments, particularly summative assessments, for paramedic students.

**Supplementary Information:**

The online version contains supplementary material available at 10.1186/s12909-023-04930-w.

## Background

There is a growing expectation that paramedics will provide a high level of care for emergency and non-emergency patients in high-pressure, time-critical environments immediately following the completion of their paramedic programme [1]. Lack of clinical competence can adversely affect patient safety and there is an increased emphasis on the quality of training and assessments that surround the clinical competence of paramedic students [2]. Performance-based examinations are an integral part of ensuring clinical competence [3]. It is important to establish paramedic clinical proficiency, at entry to practice level, as inaccurate decision-making can have significant implications for patient safety [4]. Educational institutions and regulators have a responsibility to ensure that paramedic students entering the profession are ready for independent practice [5].

The use of a variety of methods for assessment in health professions education is considered best practice [6] and in Ireland, several examination types are used by both the educational institutions and the regulatory body as part of the assessment processes for paramedics [6]. These include multiple choice question (MCQ) papers, short written answer (SWA) papers and practical assessments, using Objective Structured Clinical Examinations (OSCEs), or a variation of OSCEs and simulation-based assessments (SBAs). Simulation does and will continue to play a vital role in health professions assessment, as it permits the targeting of specific topics and skills in a safe environment [7–10]. SBAs often correlate positively with patient-related outcomes [11] and educators within the health professions continue to rely on such assessments completed in settings without direct patient contact [12].

Paramedic education programmes widely use skills sheets to support student learning and assessment. They itemise the steps in specific skills, techniques, or procedures that paramedics need to learn and master to effectively perform their job, e.g. airway management, patient assessment and incident management and triaging. They are intended to serve as a reference guide to ensure all participants adhere to the same standards and guidelines. However, a study by Martin et al. [13], suggests that using skills sheets as a scoring tool in evaluating competencies for paramedic students shows high variability and low reliability among evaluators, and questions the reliability of such a commonly used approach. In addition, a wider range of competencies such as communication, decision-making and problem-solving are required to practice as a paramedic [14]. Performance-based assessments that include these wider competencies can differentiate between levels of performance, identify achievement of pre-defined competencies, detect the ability to apply those competencies and can make accurate predictions regarding future clinical performance [4].

While summative assessment is important in ensuring that paramedic students have achieved appropriate competencies to enable them to practise safely and effectively (assessment of learning), assessment can also be considered formatively as a means to support learning [15]. Formative assessment can be viewed as assessment for learning, in which feedback plays a central role and assessment as learning, where the aim is to enhance students’ abilities to self-regulate, identify their own strengths and learning needs [16].

## Overview of current assessments

One of three recognised institutions (RIs) registered to provide paramedic training in Ireland is the National Ambulance Service College (NASC)/ University College Cork (UCC) which was accredited as an RI in October 2018 by the Pre-Hospital Emergency Care Council (PHECC) [17] who act as the national regulator for the paramedic industry. Due to the current design of the curriculum, the majority of assessments occur in the first year of the programme where, both formative and summative assessments of the students are carried out by the paramedic RIs. In addition, the regulator conducts licensing examinations at two points during the first year of the programme, through both theory and practical exams. These examinations are deemed high-stakes examinations, given that if the student fails these examinations, they fail to progress to licensing. Various types of assessment methods are typically employed including simulation, written examinations, oral examinations and reflective portfolios. One type of simulation-based assessment, called a ‘megacode’ Objective Structured Clinical Examination (OSCE) is used by both the RIs and the regulator to provide practical examinations to students. In the remaining two years of this BSc programme students move to operational exposure and participate in field-based assessments. There are fifteen full-time educators across three college sites between NASC/UCC involved in this paramedic programme. Key features of the regulator’s and NASC/UCC’s Megacode OSCE are outlined in Appendix 1.

### Research aims

International research specific to paramedic assessments is limited and while some papers describe simulation [13, 18] and simulation in the education of paramedics [18] or the assessment of paramedics in both simulation and workplace settings [19, 20] there are few exploring the opinions of educators and examiners about assessment [5, 21, 22]. Formative assessment in paramedic education has received very limited attention. Paramedic students are also assessed using MCQs and SWA examinations by both the regulator and the RIs.

The aims of this study were: (a) to seek the opinions of paramedic educators based on their experience, of assessments (theory and practical) used within their own RI and by the regulator, with a focus on assessments used in the first year of the Paramedic programme and (b) gain insights which could inform the future design of paramedic assessments. The research question asked: What are the perceptions and experiences of paramedic educators on assessment in the first year of the paramedic programme?

## Methods

As the research aimed to elicit the perceptions and experiences of paramedic educators, a qualitative, interpretivist approach was chosen. Semi-structured interviews were conducted with a purposive sample of experienced paramedic educators from across the three college sites of the NASC/UCC.

A purposive sampling technique was used for better matching of the sample to the objectives of the research, thus improving the rigour of the study and trustworthiness of the data and results [23]. We aimed to recruit both male and female participants with a range of experiences and ages. These first-hand and detailed accounts of the perceptions, actions, and roles among study participants as paramedic educators, help fulfil the criteria for credibility when conducting qualitative research [24]. A total of nine educators were selected from the fifteen full-time educators across the three NASC/UCC sites and invited to participate in semi-structured interviews. All nine educators accepted the invitation to participate by email, with an outline of the study included. Data were collected during online interviews by the Primary Investigator (PI) using MS Teams. The design of the interview guide and questions used in the semi-structured interview reflected the five phases as identified by Kallio et al. [25]. Following this approach allows other researchers to use this guide, or phased approach.

Video recordings were made using the recording function in MS Teams. The transcription function on MS Teams was used to produce initial transcripts. Transcription correction was performed by the PI, confidentiality was assured, and all data were securely transferred and stored.

### Ethics

Following ethical approval requirements, the nature and purpose of the study was carefully explained in the recruitment email. A consent form was attached and participants were made aware of their right to refuse to participate and the extent to which confidentiality would be maintained. Participants could have asked any questions before participating by contacting the PI directly by phone or email. At the beginning of each interview, the interviewer reminded participants of the terms of the consent form, including the possibility of withdrawal from the study. Participants were required to sign and date a consent form before the commencement of the interview. There was minimal anticipated risk to participants in the interviews. Interviews were conducted by the PI and no identifying data was shared outside of the research team.

### Data analysis

An inductive approach was adopted and data analysis followed Braun and Clarke’s six phases method of Thematic Analysis [26]. Using Nvivo [27], all participants responses were initially assigned codes. Codes were categorised into themes and themes were further refined in an iterative process until data saturation was considered reached. Themes were identified beyond the explicit or surface meaning of the data (i.e. at the semantic level) and further progressed to analysis at a latent level [26]. This allowed for the identification and examination of the underlying ideas, assumptions, and conceptualisations. Further, this type of thematic analysis at the latent level allows the research question to evolve through the coding process.

The PI and interviewer was a paramedic educator who had been previously employed in this recognised institution and was known to participants. At the time interviews were conducted between the first author and participants, none were in a reporting relationship with each other. This is important in terms of the positionality of the researcher within the study itself, since his involvement will have influenced and shaped the study [28], thus reflexivity was crucial to maintain the trustworthiness of the study [28]. In order to address these issues, the study design, instrument and data analysis were regularly reviewed with one of the co-authors (CS) in order to support a reflexive approach.

## Results

**Fig. 1:**
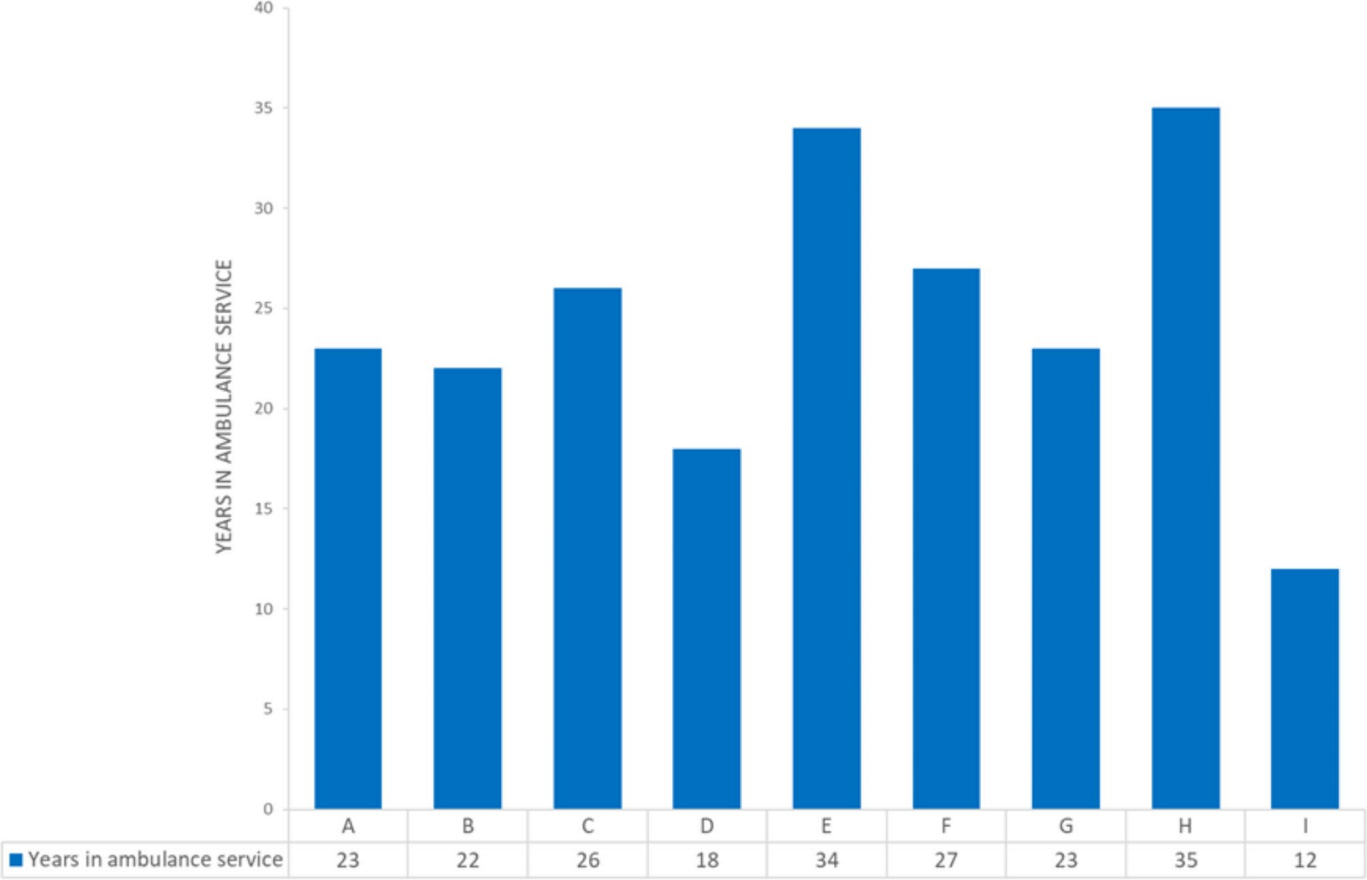
Characteristics of interview participants

Nine interviews were conducted over a 2-month period. Figure 1 summarises participants’ demographic characteristics.

Participants had worked as educators in the ambulance service for between 4 and 23 years and had spent between 12 and 35 years working in the ambulance service. There was a 6 male to 3 female gender split between all participants.

Four themes were identified, these are summarised in Table 1.

**Table 1:** Summary of themes with overviews

Theme		Explanation/overview
1	Enhancing authenticity	Improving assessments by making them more reflective of real-world practice, increasing authenticity in both practical and theory exams for RI and the regulator
2	Modifying assessment processes	Revaluate the current high-stakes examination structure conducted by the regulator, considering alternative assessment methods that better assess clinical skills.
3	Aligning PHECC and RI/University exams	Address discrepancies in pass mark requirements and examination questions between the regulator and RI/University assessments, ensuring a consistent and challenging standard that reflects real-life scenarios.
4	Utilising assessments as learning	Encourage student involvement in the assessment process through peer evaluation, question submission, and reflective practice, fostering a learning-centred approach to assessments.

### Theme 1: improving assessment by enhancing authenticity

While most participants thought assessments were fair and balanced, participants believed both the RI and regulator practical assessments could better reflect ‘real-world’ practice and be more authentic in both practical and theory exams. There were a number of ways that they identified where authenticity could be improved in various elements in both RI and PHECC exams. One participant describes the RI’s assessment, where negative marking is an integral part:

*“I think they’re very good, yeah very good because you’re not just ticking a box and its negative marking, no point in having oxygen on a patient but you haven’t recognised the major haemorrhage and he bleeds out, but you’ve ticked every box down the line, but you missed the key one at the start, so yeah.”* Participant A.

The need for more challenging environments to improve the assessment process and reflect real-world practice was suggested by some. Changing the environment for assessments would provide a more authentic approach to the student’s experience. The idea of providing an authentic environment for assessment came as a result of how students reported they benefitted from an authentic environment during formative assessments and practice.

*“Maybe outside and in different environments, you’ll get a different reaction, and we see that bringing the guys off to the fire service to get a different reaction when there’s other people involved in working around them. That would be lovely to see.”* Participant H.

Another participant described the benefits of using simulated patients instead of manikins, in an effort to replicate real-life practice.

*“For summative assessment, they absolutely have to have a person there and then allow the student to interact with them. So, while mannikins are great, I think they belong in the learning environment.”* Participant D.

To align assessment to a more authentic real-world working environment, participants identified the need to introduce an assessment where one paramedic student working alone is assessed, as paramedics are expected to be solo responders (responding to calls on their own), at times.

*“Because paramedics are being asked to act as solo responders and being on your own with a patient on the road is actually completely different when you don’t have the backup of a colleague with you…and maybe before paramedic two comes in, stop the assessment and say OK, what is your judgement now?”* Participant A.

Participants discussed the difference between the megacode OSCE presented by the RI and those presented by the regulator and while the RI provides two examiners, each assessing one student in the 2-person assessment model, the regulator provides two examiners, one to read the script and provide information to the student and the other to mark his/her performance, but not to assess the second student. It was suggested that the regulator were not assessing the work of the two-person team and therefore this was not reflective of what happens when paramedics practice in an operational capacity. The assessment only examined one person and thus lacked authenticity as both practitioners work together as a team, both should be assessed together.

*“The megacode OSCE we do have two people and we test both of them. So, we test the person who’s in charge of it and we test the person who is helping them. And the idea is that the helper, for want of a better word, doesn’t influence the decision-making on the main person. But they have to work together because that reflects their real-life environment.”* Participant E.

Participants believed that there was an appropriate mechanism within their institution’s assessment format to identify an area which formed a critical part of the examination, and if the student failed to manage this critical area at the appropriate time in the scenario, then they can fail or lose a considerable number of marks. This represents real-world practice where a patient may suffer adverse consequences because of a paramedic not doing the right thing at the right time. However, the exam conducted by the regulator was identified as lacking authenticity in this respect.

*“The megacode OSCE that we run should reflect how a real patient would respond to what’s being done by the student. It’s not real-time, but it’s the correct sequence, for example, if you don’t clear the airway, with our megacode OSCE you can’t get through that, if the airway becomes blocked, and you haven’t checked it, then you fail. If you made the same error in the PHECC one, but at the end said, oh I would have checked the airway at the beginning, you pass.”* Participant E.

While the regulator examinations include the need for the student to communicate during their practical assessment, this appears to be a simple ‘tick-box’ process. The assessment does not include a meaningful assessment of how students communicate with simulated patients, their student colleagues or anyone else involved in the assessment. Participants believed that there should be more of an emphasis on the need for students to demonstrate their ability to communicate with patients as this is a crucial part of managing patients, family members and others involved in managing a patient in the operational, non-examination setting. This perceived need for an assessment of being able to communicate appropriately was identified again across both examination types – the in-house RI’s assessments and the regulator’s assessments. One participant suggested having a standalone assessment of the student’s ability to communicate appropriately.

*“A lot of our job is actually talking to people and eliciting information from them and being able to talk to them in conversation”* Participant E.

Another participant described how the ability to simulate communicating the handover of a patient to emergency department staff, could be included as the final part of an assessment. The example includes the acronyms for relaying information in a structured fashion (ASHICE and IMIST AMBO) and therefore would replicate what would happen in reality.

*“but adding, you know, an independent part of the exam to do the ASHICE to do the IMIST AMBO for a handover to emergency department staff, I suppose an area that could be looked at for that particular element of the assessment”* Participant H.

Theory Examinations: Generally, there was overall acceptance of the two examination types used to assess the theory components of the paramedic programme. The MCQ examinations were identified as being very objective and easy to correct, however, many participants felt they could be improved to test students understanding rather than memory. The addition of clinical scenarios to MCQs was suggested by several participants.

*“What I would like to see is maybe a narrative beforehand and answer the MCQ about the narrative rather than somebody that has a good memory and that’s what I think MCQs do. So maybe an intricate type of the scenario where they’ve got to find some real detail, you know, be observant, understand, maybe the clinical condition that the patient has and then ask a series of questions about that.”* Participant H.

SWA examinations, participants believed, were a little more challenging and authentic, but some modifications could improve this assessment.

*“I think probably that PHECC short written answers are a little bit more balanced than the MCQs. With the short-written answers at least you have a number of sections on the paper. I think as well it does allow for a little bit more assessment of the depth of knowledge the student has.”* Participant F.

### Theme 2: modifying the current process of assessment

This theme considered how the regulator’s assessments were structured and if the current process of assessments could be improved. Some expressed concern that a single high-stakes assessment of clinical skills was inappropriate and questioned the process of the assessments in the final high-stakes examinations conducted by the regulator.

*“Similar to our own, I think it’s objective and its fair but it’s very high stakes and can be detrimental to someone who suffers from nerves and is having a bad day*.” Participant F.

Introducing ongoing assessments throughout the year and adding a wider variety of assessment types were considered by many participants as a way of varying the assessment process and providing more opportunities for students to demonstrate competence across a range of assessments.

*“I think continuous assessment for me would be great and it would be that the PHECC would come and have a look at some coursework, you know. I think it’s incumbent on higher education and that’s where we are, to let them see some of the project work that groups have put together.”* Participant H.

A number of participants suggested that introducing some type of continuous assessment during the paramedic programme could be recognised by the regulator and allow marks from this to contribute to the overall summative assessment result. This was particularly important given the high pass mark required in the examinations conducted by the regulator, (80% for MCQs, 70% for SWAs).

*“Is it possible to do some sort of ongoing assessment or can some of the good work we hope they have done in their institution count towards this ten minutes?”* Participant E.

There was support for changing the current assessment processes to an ongoing assessment model. It was suggested that this would align the RI and the regulator to the third-level education approach and allow for compensation across various modules of learning and placements.

*“I think there’s far too much relies on a couple of exams, whereas I think if we could spread an assessment module out, let’s say if someone was unsuccessful in one part that could bolster their results over a full year, similar to what the universities do”.* Participant G.

### Operational assessment

Some participants also discussed linking ongoing assessment to operational performance and that perhaps there should be a more inclusive type of performance assessment over a longer period of time with the aid of in-service mentors.

*“Some people that will come out and clinically their flying but they don’t actually get on with anybody are they the people that you want? You wanna take somebody on like that but they’re ticking all the boxes, but is this guy able to work on his own?”* Participant D.

The idea of using ambulance staff as mentors while students were working in ambulances, was also considered by participants as a better way to assess the students. Having a mechanism whereby ambulance staff could feedback on the students over a protracted period, was also considered.

*“I think what I would like to see is that we have people in the area who crew with the student for a week and there’s feedback on that. it’s not high stakes pass or fail assessment. It’s a formative assessment. It’s a long-term thing, we can change our behaviour when we’re being assessed for a short period of time, but when you’re with someone over a longer* period *you can’t change your behaviour like full time. So, who they’re crewed with should be able to feedback.”* Participant F.

### Audio- video (AV) recording

The utilisation of AV recording was widely identified as a significant deficit in the regulator examinations, and many believed the inclusion of AV recording could improve the quality assurance process and allow for review should an appeal be lodged by the student after the examination.

*“If I went into a room and then I believed hand on heart, that I did do something and I did it well and taken as gospel. But I may know the examiner and I may not get on well with the examiner or there could be some effect that I may perceive to happen. And I think for the interests and the safety for both examiner and examinee is the fact that there is a video which shows, this is what actually happened.”* Participant B.

Participants also discussed the benefit of using video recording for review and reflection to identify any deficits and allow the student to remediate and improve their performance should they need to re-sit the examination.

*“I think the videos are good to give both protection, to the examiners and the student, but it does allow for post-event feedback when any of the students are not successful.”* Participant G.

### Theme 3: aligning the regulator, and the RI/university examinations

Participants believed that the regulator’s practical examinations can be a tick-box exercise which do not robustly challenge the paramedic student and that some believed the requirements for a pass mark in those examinations were set at a low standard.

*“The hardest assessments our students do, are ours, not PHECC’s, they need to be coming out with a level of understanding and skill that far exceeds PHECC’s requirements.”* Participant E.

Participants believed that their RI/University assessments provided a more challenging examination to students and more closely reflected real-life scenarios and practice.

*“Our practicals, so they have to deal with whatever is life-threatening, they have to deal with that immediately if not, it’s a negative mark. If you don’t deal with the bleeding in time, you will fail. Not like PHECC’s box ticking assessment, in any order.”* Participant A.

The question of the regulator’s involvement in setting examinations, particularly the MCQ, was questioned further in relation to their pass mark requirement, and how that does not align with university pass marks. Also, the availability of the regulator’s examination questions and the lack of question bank updates or question replenishment was noted by participants. This suggests that the assessment may simply be a test of memory and a high pass mark is achievable if students know the questions. Examinations in the university, however, may be more varied and frequent, allowing for smaller focused examinations with internal and external moderation and constant test-item reviews and updates.

*“I think the pass mark is pretty high, it’s 80% currently. Uh, I think it’s, you know, when you consider it university pass rates, I think it is quite high. I think 80% in any exam is pretty high so I believe it to be a memory test.”* Participant H.

### Theme 4: opportunities to use assessment as learning

Participants highlighted examples of how students had engaged in formative assessment of their peers and had written items for summative MCQs. Participants acknowledged the role of students in their own assessments and appreciated the benefits of this. They also witnessed students recording each other on their phones while they practised skills and scenarios.

*“Students get the friend that they trust on their own phone to record them and then have them playback the assessment and have them give feedback.”* Participant A.

Some participants had asked classes to submit MCQ questions for inclusion in upcoming examinations.

*“The class gets together and puts together three or five MCQs from the weeks learning and we guarantee that a number of those questions will be in their assessment. Now the thing is, you’re not memorising them because, over the course of four weeks, that class may have asked 50 questions until they decided we’ll put these five in.”* Participant E.

Students’ participation in reflective practice following assessment was also highlighted and students were encouraged to continue this reflection when they moved to their operational roles.

*“I remind them, every time they do a call, they will have to reflect on that call and it’s just to get them to think about what did I do good there? What could I improve on and what do I take away from that?* Participant A.

## Discussion

### Principal findings

Four main themes were identified through data analysis which included: Improving assessment by enhancing authenticity, Modifying the current process of assessment, Aligning the regulator and RI/University examinations and, Opportunities to use assessment as learning.

This study identifies areas for improvement in the field of assessment and suggests there should be a different approach to the assessment of paramedic students, beyond the currently used MCQs, SWAs and Megacode OSCEs. The notion of mixed assessment methods or assessment over a more protracted period, or the use of continuous assessment, has been considered within healthcare education. Epstein [29] reminds us that all methods of assessment have strengths and intrinsic flaws, yet the use of multiple observations and several different assessment methods over time can partially compensate for the flaws in a single method [30]. Participants suggested more complex MCQs or SWAs might be beneficial. MCQ’s which include key-feature items focus on critical decisions, in particular, clinical cases might better assess processes of diagnostic reasoning [31]. Extended matching items, and several questions, all with the same long list of potential answers, can improve MCQs as they involve more complex cognitive processes [32]. The use of ‘long case’ and ‘mini-clinical-evaluation exercise’ (mini-CEX) involve candidates being observed taking a focused history and physical examination and then presenting their diagnosis and treatment plan [30].

**Fig. 2:**
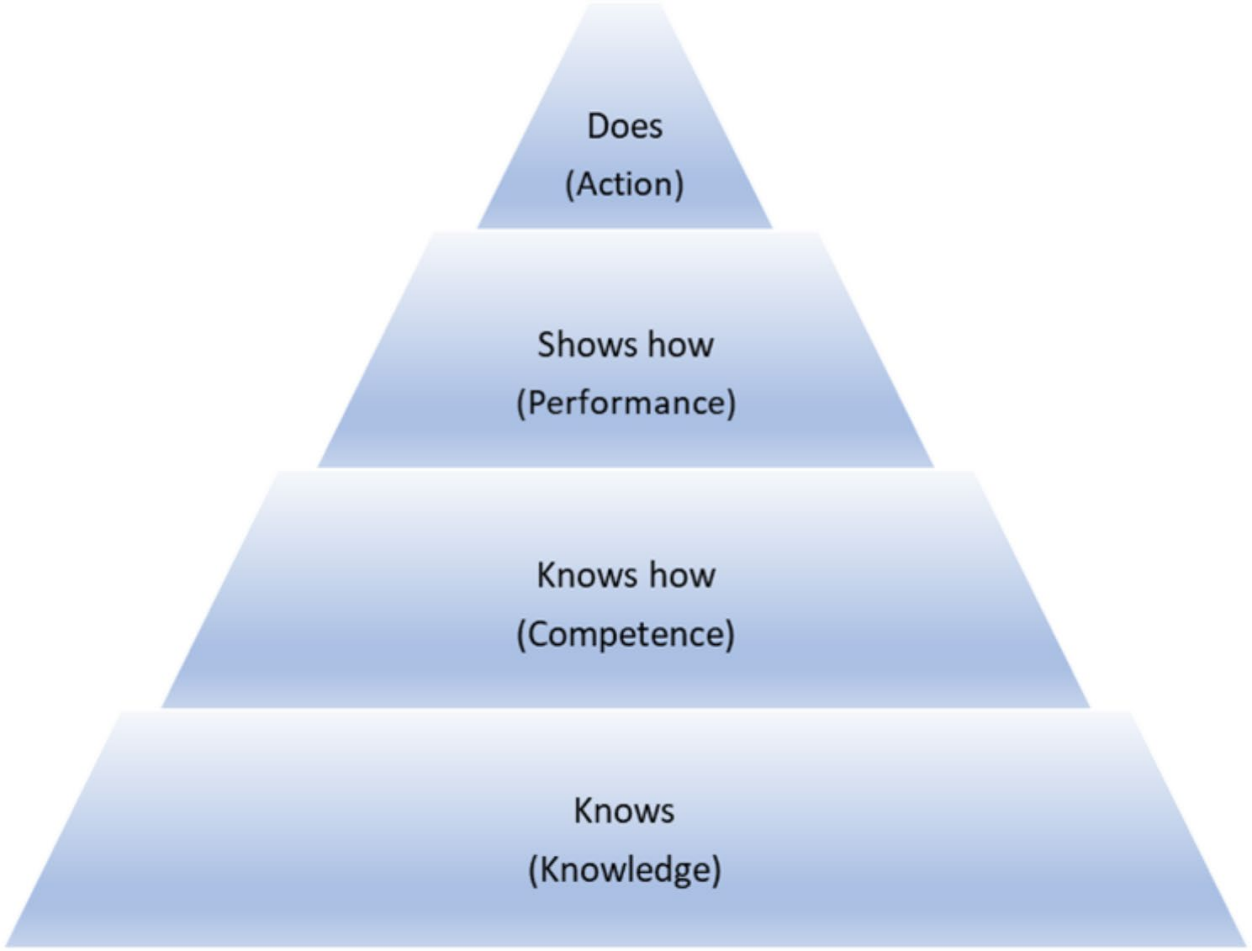
Miller’s framework for clinical assessment [3]

According to Liu [33], there are limitations with the use of OSCEs in medical education and that there is too much emphasis placed on determining if students can pass exams, an insufficient focus on whether they can perform in the role expected of them and limits on the type of cases that can be simulated. Liu describes the benefits of assessing clinical competence in the workplace and argues that these types of workplace assessments reflect the highest level of Miller’s framework for assessing competence, i.e. Action (Fig. 2) [3].

Participants suggest that the current regulator assessments could be changed to reflect more ‘real-world’ practice and fewer lower-level knowledge assessments (MCQs, SWAs). Tavares [21] suggests that authenticity refers to the degree to which the assessment context closely matches or aligns with the future clinical contexts. Ashford-Rowe et al. [34] also stress the importance of authenticity, not only in the assessment tasks prepared for students, but also for students to understand the connection between assessed skills and knowledge and the work-related application. Perhaps a shift to workplace-based assessments to include Direct observation of Procedural Skills (DOPS), Mini-Clinical Evaluation Exercise (mini-CEX) and Case-based Discussion (CbD) could provide a more real-world approach to assessing students [33].

Tavares et al. [20] described a prospective observational study analysing the assessment of student paramedics in both simulation and work-based settings. The simulation-based assessment (SBA) followed an OSCE structure involving full clinical cases from initial patient contact to the handover of care to another healthcare professional. The workplace-based assessment (WBA) reviewed samples of clinical performance during real patient encounters. Their findings suggest that the use of SBAs can be used to support evidence of clinical competence for paramedic students. This study demonstrates the benefit of using simulation with an OSCE structure as an assessment instrument to determine the competence of paramedic students.

Some issues were raised about the regulator examination in terms of the range of competencies being examined and its alignment with learning outcomes. The regulator assessment was considered by many participants to be more a test of memory than an assessment of how students might perform in the real world of practice. One example of this was a lack of focus on assessment of “handover”. In addition, the predictability of assessment content from year to year was noted. These observations suggest that a review of assessment in the regulator exam is warranted and should include a blueprinting exercise to ensure that assessment is conducted according to a replicable plan and that what is examined is mapped against learning objectives to produce a valid examination [35, 36].

Participants questioned whether the regulator should have these summative assessments at all and some argued that these should be the responsibility of the RI and University and that devolving the examinations would allow for a more varied and sustained approach to assessment. The Nursing Regulator in Ireland (NMBI), for example, approves nursing and midwifery programmes offered by the Higher Education Institutions (HEIs) which lead to registration for students [37]. The university is responsible for examinations and assessments, while awards are offered by Qualifications and Quality Ireland (QQI), who are responsible for the quality assurance of HEI programmes.

There was an identification of how students could be more involved in their own assessments by encouraging video recordings of their skills and assessment in practice to allow for personal or group critique. The notion of students developing their own MCQs was accepted as good practice and both initiatives were viewed positively by participants. This ‘assessment as learning’ allows the student to self-regulate and critically evaluate their own performances and to collaborate to develop their own shared assessment criteria. This assessment as learning may need to be given more emphasis but could result in empowering students in relation to assessment [15].

Research also indicates that when executed well, assessment as learning can enhance the results of summative assessments, benefiting learner outcomes [38]. As students become more engaged in the educational process, they gain assurance in understanding their learning objectives and the expected quality. This approach can bolster the self-assurance of learners in achieving their goals. Students begin to reflect more on their current status and their aspirations, considering the steps to achieve them. Additional advantages, like peer reviews, allow proficient students to solidify their understanding by elucidating concepts to their peers who might be struggling. This method promotes active participation and fosters autonomy in learning [16, 39]. These benefits and positive outcomes as a consequence of utilising an assessment as learning approach was strongly evidenced in the data provided by the study participants.

There are other examples of students’ participation in assessment or co-assessment such as the student-tutor consensus assessment as described by Thompson [40]. This type of assessment was developed based on previous work by Thompson [41] to introduce and validate a process of assessment, reflective practice, self-regulated learning, and sustainable assessment. These studies suggest that by introducing real-time student reflection, including recognising and learning from mistakes within practical scenario assessments, paramedic students can play an active role in decision making regarding their work and reprioritises the accountability to patient care ahead of their individual performance score.

While this cannot form part of the summative regulator assessments, it could be included in future iterations of examinations if there was on-going and continuous assessment.

### Strengths and limitations

A strength of this study is that it captures, the experiences and perspectives of a group of paramedic educators on assessments used to examine paramedic students, thus providing novel insights. The richness and diversity of the data collected for the study here support the identified themes. Further, the PI has acquired substantial and relevant experience as a paramedic educator, and the additional contributors possess both medical and health care research experience which offers a diversity of perspectives on the study data.

Nine participants were involved in the overall study and while participants in the study were representative of three sites across one RI, the findings are limited as the other two Irish RIs were not included in the study. The scope of this study was limited by the timelines imposed in completing a MSc project. It is hoped that the lack of data from the other two RIs would not impact significantly on the findings. We address the issue of transferability by providing details of the context for this research so that others may judge the relevance of the findings to their situation. However, there are circumstances when data quality can contribute more than data quantity [42, 43].

## Conclusion

The study found that participants were relatively content with their own institutional assessments but identified areas which could benefit from some improvements. The study findings suggest that if the regulator is to continue to set examinations then, assessment methods and content used by the regulator need to be strengthened to reflect real-world practice, which is of additional importance in an education environment where paramedic trainees, as “21st century educational consumer(s)” [34] increasingly seek robust, relevant and authentic work-related competencies and skills. These findings also raise the question of whether or not the regulator should continue to host the examinations. The introduction of continuous assessment, assessment as learning, the introduction of a communications assessment (either within the examination or as part of continuous assessment) and the devolvement of all assessments and examinations to the RI/university partnership could address concerns identified by those involved in the education of Irish paramedics and improve the quality of assessments.

### Electronic supplementary material

Below is the link to the electronic supplementary material.

Supplementary Material 1

## Data Availability

The datasets used and/or analysed during the current study available from the corresponding author on reasonable request.

## List of abbreviations

RIs: Recognised Institutions
PHECC: Pre-Hospital Emergency Care Council
MCQ: multiple choice question
SWA: short written answer
OSCEs: Objective Structured Clinical Examinations
NASC: National Ambulance Service College
UCC: University College Cork
SREC: Social Research Ethics Committee
PI: Principle Investigator
MS: Microsoft
