# Supplementary material for: Perceptions of paramedic educators on assessments used in the first year of a paramedic programme: a qualitative exploration
Source: BMC Med Educ. 2023 Dec 12;23:952. doi: 10.1186/s12909-023-04930-w (PMC10717895; doi:10.1186/s12909-023-04930-w)
Supplement: Supplementary file 1 — Supplementary Material 1 [file 12909_2023_4930_MOESM1_ESM.docx]

*S1*

*Megacode OSCE: similarities and differences between PHECC’s and NASC/UCC’s*

| Megacode OSCE | PHECC Megacode OSCE | NASC/UCC Megacode OSCE |
| --- | --- | --- |
| Two students used in examination | Yes | Yes |
| Student 1 (lead) assessed | Yes | Yes |
| Student 2 (2^nd^ practitioner) assessed | No | Yes |
| Two Examiners in a room with students/candidates | Yes | Yes |
| Role of Examiner 1 | Assesses Student 1 | Assesses Student 1 |
| Role of Examiner 2 | Narrator reads scenario, provides information | Assesses Student 2 |
| Discusses students’ performance with other examiner, at end of each examination | No. Discussion between examiners is not permitted | Yes. Discussion between examiners is encouraged |
| Multiple selection of scenarios^[[1]](#footnote-1)^ | No | Yes |
| Use of Audio-Visual Recording for Quality Assurance or Review as part of an appeal by a student | No | Yes |
| Used as a summative assessment | Yes, for practitioner licensing | Yes, in-house |
| Student can re-sit Megacode | Yes, following remediation | Yes, but limited to four recorded fails (in-house) across all assessments |

1. This means that PHECC only has up to eight scenarios set for their examinations, meaning students usually know what these eight scenarios are. NASC/UCC however, select exams from a suite of assessments and are not restricted to only eight, meaning students have less chance in knowing what type of scenario will be used for the assessment. [↑](#footnote-ref-1)
